# Conversion surgery after lenvatinib treatment for anaplastic thyroid carcinoma: a case report

**DOI:** 10.1186/s40792-023-01619-6

**Published:** 2023-03-15

**Authors:** Haruhiko Yamazaki, Katsuhiko Masudo, Sachie Kanada, Yoshiaki Inayama, Hiroyuki Hayashi, Yu Fujii, Yasushi Rino

**Affiliations:** 1 Department of Breast and Thyroid Surgery, Yokohama City University Medical Center, 4-57 Urafunecho, Minami-Ku, Yokohama, Kanagawa 232-0024 Japan; 2 Department of Diagnostic Pathology, Yokohama City University Medical Center, 4-57 Urafunecho, Minami-Ku, Yokohama, Kanagawa 232-0024 Japan; 3 Department of Pathology, Yokohama Municipal Citizen’s Hospital, 1-1 Mitsuzawanishicho, Kanagawa-Ku, Yokohama, Kanagawa 221-0855 Japan; 4 Department of Surgery, Yokohama City University School of Medicine, 3-9 Fukuura, Kanazawa-Ku, Yokohama, Kanagawa 236-0004 Japan

**Keywords:** Anaplastic thyroid carcinoma, Conversion surgery, Lenvatinib

## Abstract

**Background:**

Anaplastic thyroid carcinoma (ATC) is the most aggressive form of thyroid carcinoma. Lenvatinib, a multikinase inhibitor, is rarely used in preoperative settings due to adverse effects including delayed wound healing and fistula formation. Herein, we report the use of lenvatinib treatment prior to conversion surgery for the treatment of ATC.

**Case presentation:**

A 71-year-old woman was referred to our hospital with suspected thyroid cancer with recurrent laryngeal nerve invasion and cervical lymph node metastasis based on the results of ultrasonography. Computed tomography demonstrated the presence of a thyroid tumor invading the trachea and esophagus with no evidence of distant metastasis. Fine needle aspiration of the left cervical lymph node indicated the lymph node metastasis of ATC. As the tumor had widely invaded the trachea and esophagus, unresectable ATC was diagnosed and treatment with lenvatinib was initiated at a dose of 24 mg/day. On day 13 of lenvatinib treatment, the primary tumor and lymph node metastases demonstrated a partial response to therapy. As the tumor was now considered resectable, the decision was made to perform conversion surgery. Total thyroidectomy and left lateral neck node dissection were performed 7 days after the withdrawal of lenvatinib. The patient was discharged on postoperative day 5 with no complications. Histopathological examination demonstrated that the tumor contained the component of papillary thyroid carcinoma, squamoid ATC cells, and granulation tissue. In areas of granulation tissue, atypical cells with spindle-shaped or polygonal morphology, pyknotic nuclei, and scant cytoplasm were observed. Immunohistochemically, these cells were positive for cytokeratin AE1/AE3, TTF-1, and p53 and negative for thyroglobulin and PAX8. Therefore, the areas of granulation tissue observed within tumor samples were also considered ATC that were affected by lenvatinib treatment. In total, approximately 50% of resected tumor comprised ATC, and 70% of them had been changed to granulation tissue.

**Conclusions:**

The findings in the present case indicate that lenvatinib may have significant antitumor effects in preoperative settings. Lenvatinib may represent a promising candidate therapy for unresectable ATC by increasing tumor resectability.

## Background

Anaplastic thyroid carcinoma (ATC) is the most aggressive form of thyroid carcinoma [1]. In the revised Japanese clinical practice guidelines on the management of thyroid tumors, multidisciplinary treatment comprising radiation therapy, chemotherapy, and molecular-targeted therapy are recommended for ATC [2]. In contrast, the latest American Thyroid Association Guidelines recommend screening for *BRAF* mutations using immunohistochemistry or molecular testing at the time of diagnosis [3]. There have been case reports of patients with ATC undergoing curative resection after neoadjuvant therapy with *BRAF* and *MEK* inhibitors [4] or entrectinib [5]. However, targetable gene mutations may not always be identified in cases of ATC [5, 6]. Lenvatinib, a multikinase inhibitor, has recently been approved for the treatment of ATC in Japan [2] and can be used in cases without targetable gene mutations. However, lenvatinib is rarely used in preoperative settings due to adverse effects including delayed wound healing and fistula formation [7]. Herein, we report the use of lenvatinib treatment prior to conversion surgery for the treatment of ATC.

## Case presentation

**Fig. 1:**
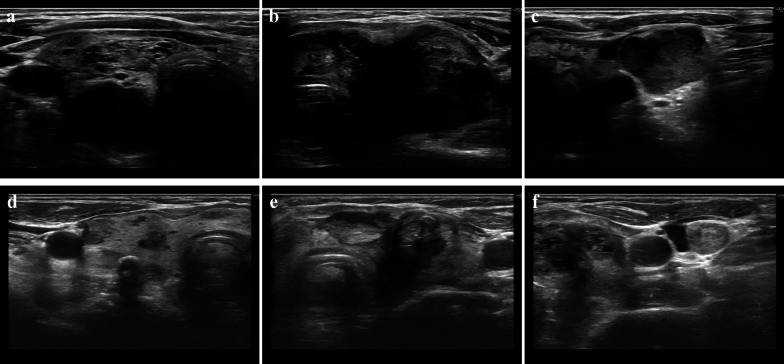
Ultrasonography before and after lenvatinib treatment. **a**–**c** Ultrasonography revealed a hypoechoic mass in the left lobe of the thyroid and lymph node metastases in the right paratracheal and left cervical regions. **d**–**f** A hypoechoic mass in the left lobe of the thyroid and lymph node metastases in the right paratracheal and left cervical regions shrank after lenvatinib treatment

**Fig. 2:**
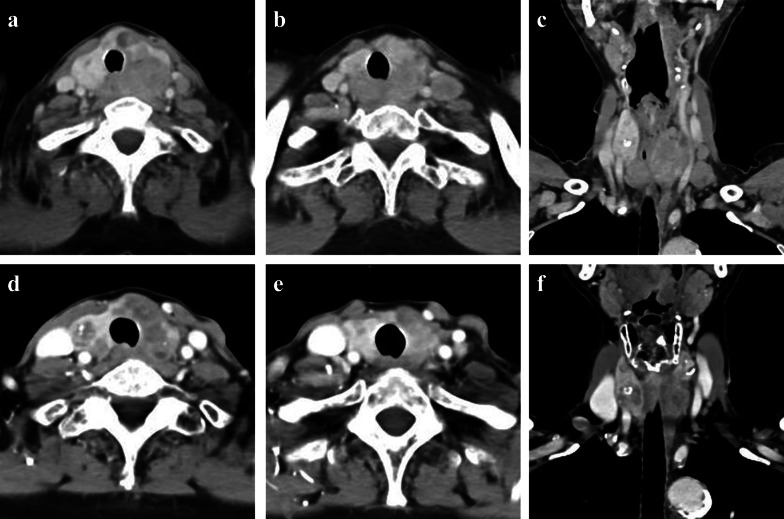
Computed tomography before and after lenvatinib treatment. **a**–**c** Computed tomography demonstrated the presence of a thyroid tumor measuring 4.3 × 3.3 cm and left cervical lymph node metastasis measuring 1.9 × 1.6 cm. The thyroid tumor widely invaded the trachea and esophagus. There was no evidence of distant metastasis. **d**–**f**. The thyroid tumor and left cervical lymph node metastasis shrank to 3.3 × 2.1 cm and 0.8 × 0.8 cm, respectively, after lenvatinib treatment

**Fig. 3:**
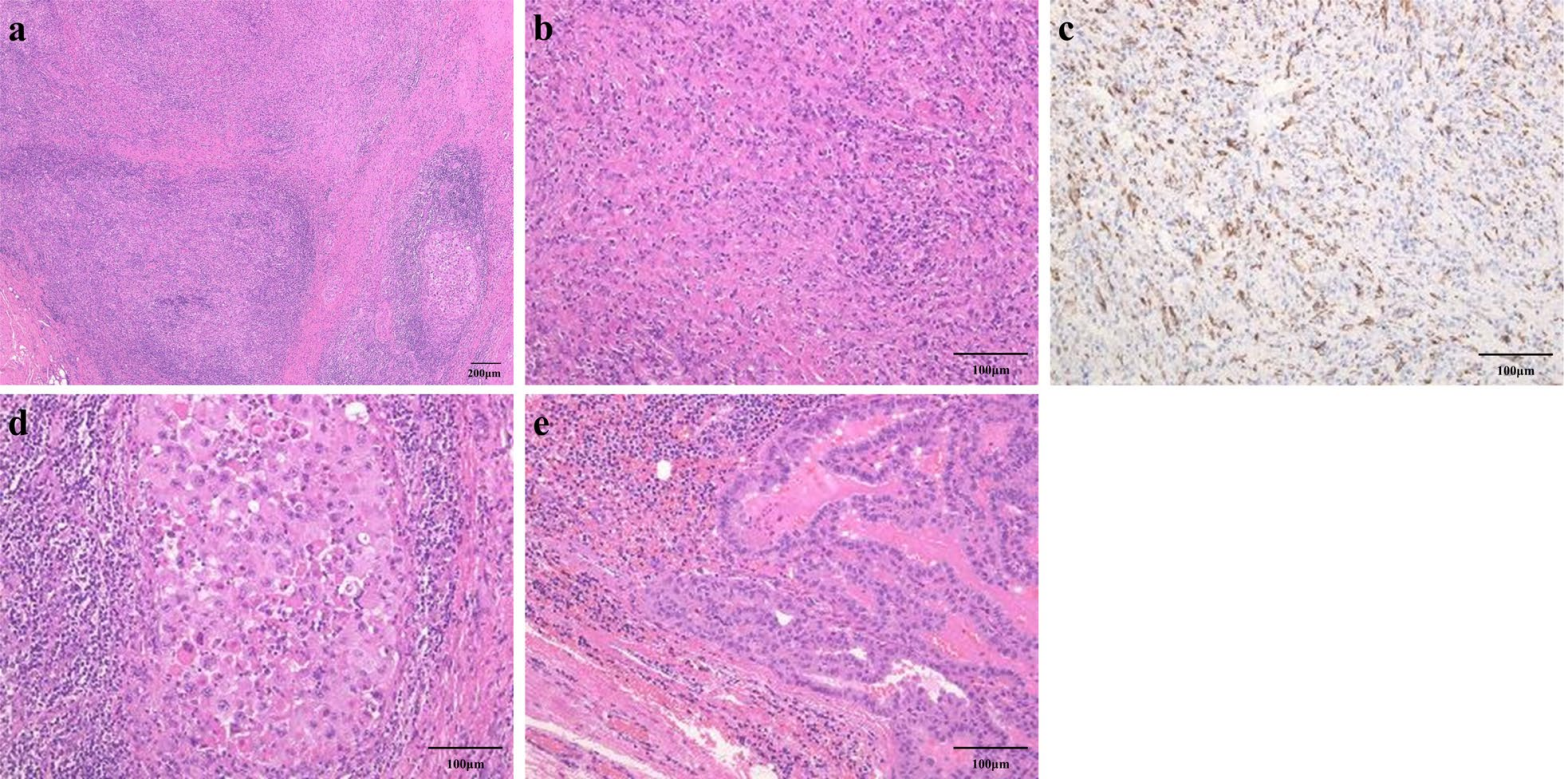
Histopathological examination of surgical specimens. Low-power view of the anaplastic thyroid carcinoma (ATC) component (**a**). Inflammatory granulation tissue is observed on the left side, whereas highly atypical squamoid ATC cells are still remaining on the right. The granulation tissue contains many small spindle cells with degenerative pyknotic nuclei (**b**) which are distinctly positive for cytokeratin AE1/AE3 (**c**). High-power view of the viable ATC cells (**d**). Papillary thyroid carcinoma is observed on the periphery of the ATC (**e**).

**Fig. 4:**
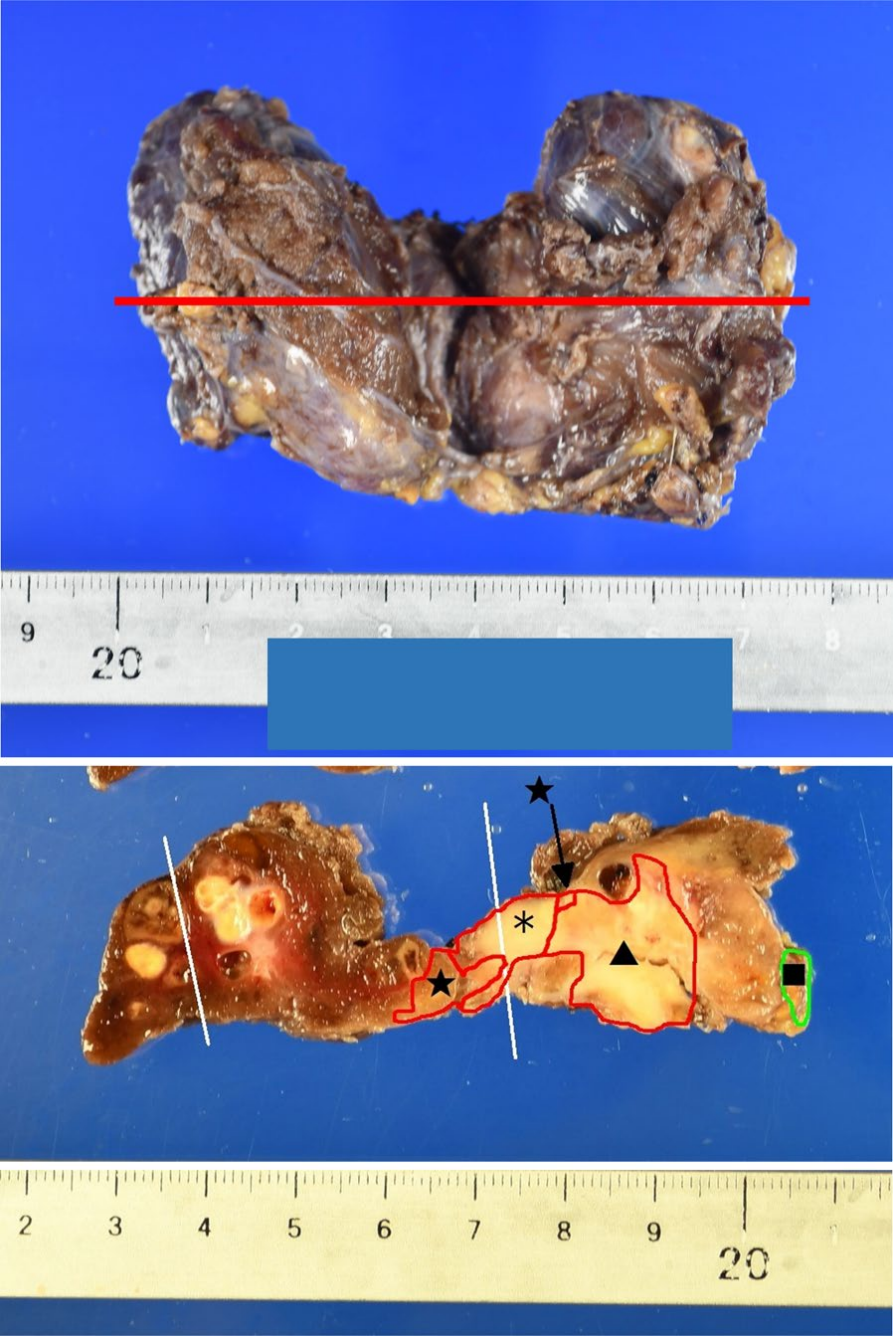
Distribution of tumor. In total, approximately 50% of resected tumor comprised anaplastic thyroid carcinoma (ATC), and 70% of them had been changed to granulation tissue. ★: ATC, *: granulation with degenerative ATC, ▲: papillary thyroid carcinoma, ■: parathyroid gland

**Fig. 5:**
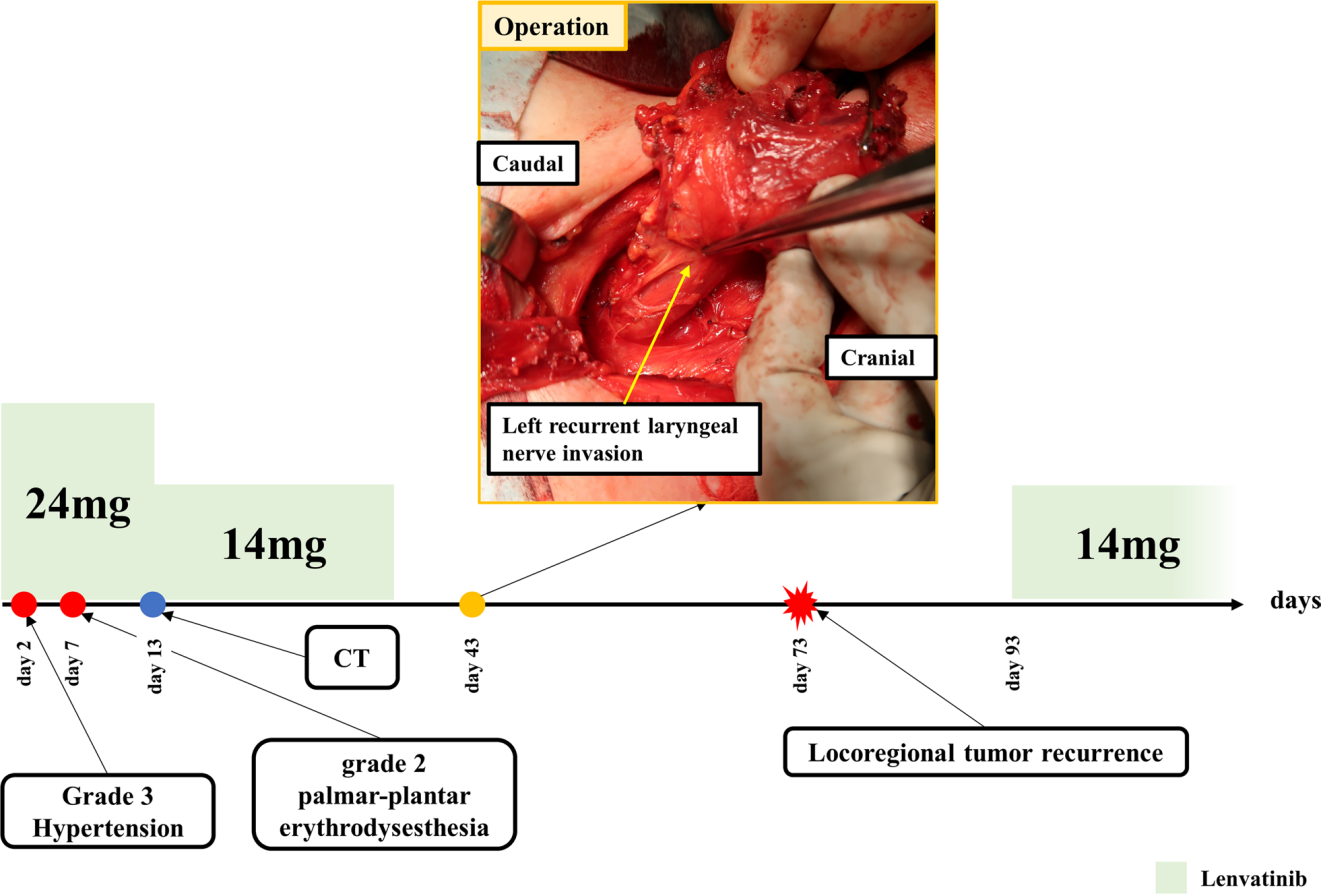
Treatment and clinical course

A 71-year-old woman consulted her physician complaining of hoarseness. She was referred to our hospital with suspected thyroid cancer with recurrent laryngeal nerve invasion and cervical lymph node metastasis based on the results of ultrasonography. A diffuse goiter and cervical lymphadenopathy were noted on examination. Blood testing demonstrated a white blood cell count level of 5440 /μL, thyroid stimulating hormone level of 0.44 µIU/ml, free triiodothyronine level of 3.01 pg/ml, free thyroxine level of 1.05 ng/ml, thyroglobulin level of 285 ng/ml, and anti-thyroglobulin antibody level of less than 10 IU/ml. Ultrasonography revealed a hypoechoic mass in the left lobe of the thyroid and lymphadenopathy suspicious for metastasis in the right paratracheal and left cervical regions (Fig. 1a–c). Computed tomography (CT) demonstrated the presence of a thyroid tumor invading the trachea and esophagus with no evidence of distant metastasis (Fig. 2a–c). Fluorodeoxyglucose positron emission tomography revealed significant uptake by the thyroid tumor and a left cervical lymph node without uptake in distant organs. Fine needle aspiration of the left cervical lymph node indicated the lymph node metastasis of ATC. Biopsy of the left cervical lymph node was performed to screen for *RET* mutations using the Oncomine Dx Target Test (Thermo-Fisher, USA). The sampled lymph node tissue was negative for *RET* mutations; however, a *BRAF* mutation was identified. As the tumor had widely invaded the trachea and esophagus, unresectable ATC was diagnosed and treatment with lenvatinib was initiated at a dose of 24 mg/day. Since there was the fatal risk of fistula formation, we had fully informed the patient about serious adverse events before starting lenvatinib. Furthermore, we decided to perform CT earlier to investigate whether fistula formation occurred. On day 2 of lenvatinib administration, the patient developed grade 3 hypertension and treatment with a calcium channel blocker and an angiotensin receptor blocker was initiated accordingly. On day 7 of lenvatinib treatment, the patient developed grade 2 palmar–plantar erythrodysesthesia that was managed with managed conservatively. On day 13 of lenvatinib treatment, the primary tumor and lymph node metastases demonstrated a partial response to therapy (Figs. 1d–f, 2d–f). As the tumor was now considered resectable, the decision was made to perform conversion surgery. On day 14 of lenvatinib administration, the patient was discharged and the lenvatinib dose was reduced to 14 mg/day to reduce the risk of further adverse events (AEs) such as fistula formation. Total thyroidectomy and left lateral neck node dissection were performed 7 days after the withdrawal of lenvatinib. The operative duration was 163 min with minimal blood loss. As the tumor encased the left recurrent laryngeal nerve, we performed combined resection of the tumor and left recurrent laryngeal nerve. As the tumor was found to be invading the first tracheal cartilage, tracheal shaving was performed to preserve the trachea. On the other hand, there was no obvious invasion into the esophagus and dissection was easy.　The patient was discharged on postoperative day 5 with no complications. Histopathological examination demonstrated that the tumor contained the component of papillary thyroid carcinoma (PTC), squamoid ATC cells, and granulation tissue (Fig. 3a, e). Those squamoid ATC cells with prominent nuclear atypia (Fig. 3d) were weakly positive for cytokeratin AE1/AE3, thyroid transcription factor 1 (TTF-1), and thyroglobulin. Furthermore, PAX8 staining was negative and p53 staining was positive. These results were considered representative of ATC. In areas of granulation tissue, atypical cells with spindle-shaped or polygonal morphology, pyknotic nuclei, and scant cytoplasm were observed (Fig. 3b). Immunohistochemically, these cells were positive for cytokeratin AE1/AE3 (Fig. 3c), TTF-1, and p53 and negative for thyroglobulin and PAX8. Given the above findings, the areas of granulation tissue observed within tumor samples were also considered ATC that were affected by lenvatinib treatment. The tracheal invasion area also had similar findings that contained squamoid ATC cells and granulation tissues. In total, the proportion of ATC component was approximately 50% among the whole tumor. Additionally, we diagnosed that 70% of ATC component had been changed to granulation tissue (Fig. 4). Postoperative radiotherapy was planned; however, lenvatinib was restarted at 30 days postoperatively due to tumor recurrence in the subcutaneous tissues and lymph nodes of the right neck. The summary of treatment and clinical course is shown in Fig. 5.

## Discussion

Lenvatinib is rarely used in preoperative settings and the effectiveness of neoadjuvant therapy for thyroid carcinoma remains uncertain. However, the effectiveness of neoadjuvant therapies comprising tyrosine kinase inhibitors or inhibitors of angiogenesis has been reported in other types of unresectable cancer [8, 9]. Tsuboi et al. reported the first preoperative use of lenvatinib for advanced PTC [10]. In their case, the lenvatinib withdrawal period was 17 days and the patient was discharged 14 days after surgery without postoperative complications [10]. Subsequently, Iwasaki et al. reported a case series of preoperative lenvatinib for advanced thyroid carcinoma including two patients with ATC, with the lenvatinib withdrawal period ranging from four to ten days [11]. In our case, the lenvatinib withdrawal period was seven days, which was based on previous reports [10, 11], and no postoperative complications occurred. As the half-life of lenvatinib is reported to be 35.4 h [12], a withdrawal period of seven days is likely sufficient in preoperative settings.

The combination of dabrafenib (*BRAF* inhibitor) and trametinib (*MEK* inhibitor) was the first targeted therapy regimen approved for ATC [13]; however, these agents have not been approved for use in Japan. Limited data indicate poor response rates in cases of ATC treated with paclitaxel, a microtubule-stabilizing drug [14, 15]. Tumor response to treatment is typically the most important factor in determining subsequent treatment in patients with unresectable disease as tumors may become resectable as tumor size decreases. Accordingly, lenvatinib may have utility as a neoadjuvant therapy for thyroid cancer given its significant antitumor effect [7]. The tumor in the present case was deemed to be unresectable prior to lenvatinib treatment; however, the tumor decreased in size and become resectable after just 2 weeks of lenvatinib treatment.

The timing of lenvatinib initiation, particularly with the aim of preventing AEs, has previously been discussed [16]. However, long-term use of lenvatinib therapy is unlikely to be required preoperatively, thereby limiting the risks of lenvatinib-associated AEs. In the present case, all AEs were manageable and lenvatinib therapy was not immediately restarted given the successful resection of almost all tumors.

Regarding the use of tyrosine kinase inhibitors in preoperative settings, Huag et al. reported the efficacy of anlotinib in 13 patients with locally advanced thyroid carcinoma [17]. Anlotinib was shown to antitumor activity when used as a neoadjuvant therapy, with the majority of patients subsequently undergoing R0 or R1 resection. [17]. As R0/R1 resection status likely impacts on prognosis in patients with ATC [18], preoperative use of tyrosine kinase inhibitors may have utility in increasing survival.

There are limited reports of pathological assessments after targeted therapy in thyroid carcinoma. Katoh et al. reported a case of locally advanced PTC that underwent resection after lenvatinib treatment where tumor cells were found to be completely replaced by necrotic tissue and fibrosis on pathological examination [19]. Similarly, extensive necrosis and fibrosis on histological examination following lenvatinib therapy was reported in a separate case [20]. Similar pathological findings have also been reported following the use of targeted therapy including entrectinib and the combination of dabrafenib and trametinib [4, 5]. Accordingly, necrosis and fibrosis may be treatment effects of targeted therapy in thyroid carcinoma. In the present case, the areas of granulation tissue in tumor samples were considered to be composed of cancer cells that had undergone nuclear destruction and cytoplasmic degeneration. These areas of tumor on histological analysis may indicate the effect of lenvatinib as a preoperative treatment in ATC.

## Conclusions

The findings in the present case indicate that lenvatinib may have significant antitumor effects in preoperative settings. Furthermore, we observed no postoperative complication after withdrawing lenvatinib for 7 days prior to surgery. Lenvatinib may represent a promising candidate therapy for unresectable ATC by increasing tumor resectability.

## Data Availability

The datasets used and/or analyzed during the current study are available from the corresponding author upon reasonable request.

## Abbreviations

AE: Adverse event
ATC: Anaplastic thyroid carcinoma
CT: Computed tomography
PTC: Papillary thyroid carcinoma
TTF-1: Thyroid transcription factor 1
